# Supplementary material for: The Genetic Landscape of Mitochondrial Diseases in Spain: A Nationwide Call
Source: Genes (Basel). 2021 Oct 9;12(10):1590. doi: 10.3390/genes12101590 (PMC8535857; doi:10.3390/genes12101590)
Supplement: Supplementary file 1 [file genes-12-01590-s001.zip › Supplementary Table S1.pdf]

General information

Record ID ______________________

NHC ______________________

Hospital ______________________

Date of birth ______________________

Place of birth ______________________

Sex
- ◯ Male
- ◯ Female

Ethnics
- ◯ Caucasian
- ◯ Subsaharian african
- ◯ Southamerican
- ◯ Asian
- ◯ Gypsy

Affected relatives
- ◯ Yes
- ◯ No

Consanguinity
- ◯ Yes
- ◯ No

Genetic inheritance
- ◯ Maternal
- ◯ Recessive
- ◯ Dominant
- ◯ Unknown

Age of onset (specify months or years) ______________________

Death
- ◯ Yes
- ◯ No

Specify cause of death ______________________

Clinical characteristics

Diagnostic

- ◯ Alpers
- ◯ Ataxia
- ◯ Barth
- ◯ Cardiomyopathy
- ◯ Carnitine disorders
- ◯ Cartilage-hair hypoplasia
- ◯ Congenital weakness
- ◯ Cramps
- ◯ Deafness
- ◯ Deafness-dystonia
- ◯ Diabetis
- ◯ DIDMOAD
- ◯ Dystonia
- ◯ Encephalopathy
- ◯ Fatigue and exercise intolerance
- ◯ Friedreich ataxia
- ◯ Gastrointestinal
- ◯ HAM (hearing loss, ataxia, myoclonus)
- ◯ Hepatic
- ◯ Hypoglicemia
- ◯ Infantile CNS
- ◯ Kearns- Sayre
- ◯ Lebers-optic (LHON)
- ◯ Leygh syndrome
- ◯ Leukodystrophy
- ◯ Lipomatosis
- ◯ MELAS (Mitochondrial Encephalomyopathy, Lactic Acidosis, Stroke)
- ◯ MERFF (Myoclonic Epilepsy, Ragged Red Fibers)
- ◯ MILS (Leigh syndrome, maternal inheritance)
- ◯ MLASA (lactic acidosis, Sideroblastic Anemia)
- ◯ MNGIE (Myopathy and external ophtalmoplegia, Neuropathy, Gastro-Intestinal, Encephalopathy)
- ◯ Myalgias
- ◯ Myoglobinuria
- ◯ Myopathy syndrome
- ◯ NARP/MILS (Neuropathy, Ataxia, Retinitis Pigmentosa)
- ◯ Neuropathy
- ◯ Ophtalmoplegia External (PEO)
- ◯ Pancreas
- ◯ Parkinson's
- ◯ Pearson's
- ◯ Rhabdomyolysis
- ◯ SANDO (Sensory Ataxic Neuropathy Dysarthria, Ophthalmoparesis)
- ◯ Spastic Paraparesis
- ◯ Spinal muscular atrop
- ◯ Toxic (drug)
- ◯ Other
- ◯ Unknown

Mitochondrial disease criteria (Morava et al 2006)

[Attachment: "criterios clínicos Morava Neurology 2006.pdf"]

Mitochondrial disease criteria

- ◯ 1: mitochondrial disorder unlikely
- ◯ 2-4: possible mitochondrial disorder
- ◯ 5-7: probable mitochondrial disorder
- ◯ 8-12: definitive mitochondrial disorder

DISEASE ONSET SYMPTOMS

Neuromuscular disorders symptoms

- ◯ Yes
- ◯ No

Neuromuscular disorders

- ☐ Cardiac arrhythmia
- ☐ cardiomyopathy
- ☐ Exercise intolerance
- ☐ Hypotonia
- ☐ Muscle wasting
- ☐ Muscle weakness
- ☐ Myalgia/muscle pain
- ☐ Myoglobinuria
- ☐ Peripheral neuropathy
- ☐ Respiratory impariment
- ☐ Other

If other neuromuscular disorder symptoms, please specify

____

Eyes symptoms

- ◯ Yes
- ◯ No

Eyes

- ☐ Cataract
- ☐ Eyelid ptosis
- ☐ Ophtalmoparesis
- ☐ Optic neuropathy
- ☐ Retinopathy
- ☐ Visual impairment
- ☐ Other

If other eyes symptoms, please specify

____

Brain and movement disorders symptoms

- ◯ Yes
- ◯ No

Brain and movement disorders

- ☐ Ataxia
- ☐ Cerebellar signs
- ☐ Consciousness disturbance
- ☐ Dementia
- ☐ Dyskinesia
- ☐ Dystonia
- ☐ Epilectic seizures
- ☐ Hearing loss
- ☐ Microcephaly
- ☐ Migraine
- ☐ Motor-neuron disease
- ☐ Myoclonus
- ☐ Nystagmus
- ☐ Parkinsonisms
- ☐ Psychiatric symptoms/abnormal behaviour
- ☐ Neurological regression
- ☐ Psychomotor retardation
- ☐ Pyramidal signs
- ☐ Status epilepticus
- ☐ Stroke-like episodes
- ☐ Tremor
- ☐ Unspecified abnormal movements
- ☐ Unspecified abnormal eye movements
- ☐ Unspecified epileptic seizures
- ☐ Dysautonomic symptoms
- ☐ Bulbar dysfunction
- ☐ Other

If other brain and movement disorders, please specify ________________________________

Gastrointestinal and endocrinopathy symptoms

- ◯ Yes
- ◯ No

Gastrointestinal and endocrinopathy

- ☐ Diabetis mellitus
- ☐ Failure to thrive
- ☐ Gastrorintestinal dysmotility
- ☐ Hepatopathy
- ☐ Hypogonadism
- ☐ Hypoparathyroidism
- ☐ Hypothyroidism
- ☐ Kidney involvement
- ☐ Short stature
- ☐ Swallowing impairment
- ☐ Vomiting
- ☐ pancreatic insufficiency
- ☐ Other

If other gastrointestinal symptoms, please specify

Blood abnormalities symptoms

- ◯ Yes
- ◯ No

Blood abnormalities

- ☐ Anaemia
- ☐ Increment of ALT/AST
- ☐ Increment of CK
- ☐ Neutropenia
- ☐ Pancytopenia
- ☐ Abnormal clotting factors
- ☐ Others

If other blood abnormalities, please specify ________________________________

Cutaneus involvement symptoms

- ◯ Yes
- ◯ No

General appearance and cutaneous involvement

- ☐ Acrocyanosis
- ☐ Dysmorphic features
- ☐ Hirsutism
- ☐ Multiple lipomatosis
- ☐ Hair abnormalities
- ☐ Abnormal pigmentation
- ☐ Other

If other cutaneuous symptoms, please specify ________________________________

The Newcastle Mitochondrial Scale

Please select one of the options
◯ Paediatric patient
◯ Adult patient

If paediatric, choose one option
◯ NPMDS 0-24 months
◯ NPMDS 2-11 years
◯ NPMDS 12-18 years

THE NEWCASTLE PAEDIATRIC MITOCHONDRIAL DISEASE SCALE

[Attachment: "NPMDS escala 0 - 24 meses.pdf"]

NPMDS date ________________________________

Section I: Current Function (preceding 2 week period)

Vision
◯ Normal. No parental concerns
◯ MIld. Limited eye or head movement to large objects or parental face in visula field
◯ Moderate. No response to large objects or parental face in the visual field
◯ Servere. No response to light

Hearing
◯ Normal
◯ MIld. Body, head or eye movement only to loud noise
◯ Moderate. No reaction to loud noise
◯ Severe. No hearing (even with aid)

Communication (assessed with appropriate regard for age)
◯ Normal. Age appropriate communication
◯ Mild. Delayed development of communication
◯ Moderate. Communication unintelligible to parents or completely reliant on non-verbal communication
◯ Severe Not communicating effectively in any form.

Feeding
◯ Normal
◯ Mild. Difficulties in sucking/ coughing/ anorexia/wheezy with feeds or occasional choking
◯ Moderate: Supplementary enteral feeding or recurrent aspiration pneumonia
◯ Severe. elusive enteral feeding (gastrostomy/NG tube). Nil by mouth

Mobility
◯ Normal. No concerns. Age appropriate mobility
◯ Mild. Clumsy age appropriate mode of mobility
◯ Moderate. Mobile but through age inappropriate mode
◯ Severe. Immobile

Section I score ________________________________

Section II: System Specific Involvement (preceding 6 month period)

Seizures
- ◯ None
- ◯ Mild. Myoclonic or absence seizures only or < 1 generalised tonic-clonic seizure/month
- ◯ Moderate. >5 generalized tonic-clonic seizures/month or >20 absence or myoclonic seizures/month
- ◯ Severe, Status epilepticus or intractable seizures

Encephalopathy
- ◯ NOne
- ◯ Mild. Abnormal sleepiness/lethargy. Waking only for feeds
- ◯ Moderate. Recurrent episodes of mild encephalopathy (>2/years)
- ◯ Severe. Life threatening encephalopathy-requires artificial ventilation

Gastrointestinal
- ◯ Normal
- ◯ Mild. Constipation or unexplained vomiting/diarrhoea >3 week
- ◯ Moderate. Severe constipation (no relief with laxative treatment) or unexplained vomiting/diarrhoea every day or surgical intervention for dysmotility
- ◯ Severe. Malbsorption/Failure to thrive

Endocrine
- ◯ Normal
- ◯ MIld. Biochemical evidence of impaired function
- ◯ Moderate. Endocrine failure requiring replacement therapy
- ◯ Severe. Endocrine decompensation (e.g. diabetic ketoacidosis, addisonian crisis)

Respiratory
- ◯ Mild. Abnormal respiratory pattern not requiring therapy/hospitalisation
- ◯ Moderate. Abnormal respiration requiring osygen flow or hospitalisation but not ventilation
- ◯ Severe. Abnormal respiration requiring artificial ventilation

Cardiovascular (over preceding 12 months)
- ◯ Normal
- ◯ Mild. Asymptomatic ECG change
- ◯ Moderate. Abnormal echocardiogram (e. g. cardiomegaly or sustained/ symptomatic arrhythmia on ECG)
- ◯ Severe. Decompensated cardiomyopathy or requiring pacing device/debibrillator/ablation

Renal
- ◯ Normal
- ◯ Mild. Impaired dunction but no change in diet or therapy required
- ◯ Moderate. Impaired function requiring restricted protein diet
- ◯ Severe. Failure requiring transplant/ dialysis

Liver

- ◯ Normal
- ◯ Mild. MIldly impaired Liver Function Tests (LFTs). Normal albumin and coagulation. No symptoms of hepatic failure
- ◯ Moderate. Impaired LFTs with symptoms (e.g. jaundice, coagulation anomalities, oedema)
- ◯ Severe. Failure requiring hospitalisation and/ or transplantation

Blood

- ◯ Normal
- ◯ Mild. Anaemia only
- ◯ Moderate. Asymptomatic pancytopenia
- ◯ Severe. Pancytopenia requiring regular transfusion/ transplantation

Section II score ________________________________

Section III: Current Clinical Assessment (at the time of assessment)

Growth (weight) over preceding 6 months

- ◯ Normal. Following normal growth trajectory
- ◯ Mild. Weight less than second centile but growing parallel to it
- ◯ Moderte. Weight crossing one centile
- ◯ Severe. Weight crossin >=2 centiles or less than second centile with divergent trajectory

Development over preceding 4 months ________________________________

Vision

- ◯ Normal
- ◯ Mild. IMpaired fixatin and tracking
- ◯ Moderate. Impaired fixation and/or tracking of familiar faces
- ◯ Severe. No response to light or registered blind

Ptosis and eye movement

- ◯ Normal
- ◯ Mild. Gaze evoked nystagmus or unilateral ptosis or imparied eye movement at extremities
- ◯ Moderate. Intermittent nystagmus at rest or bilateral ptosis not obscuring pupils or restriction of >50% eye movement
- ◯ Severe. Continuous nystagmus at rest or bilateral ptosis obscuring pupils or only a flicker of eye movement.

Myopathy

- ◯ Normal
- ◯ Mild. Mild symmetrical weakness of hip and/or shoulder girdle only
- ◯ Moderate. Moderate symmetrical weakness (proximal> distal) limiting functional movement
- ◯ Severe. Wheelchair/carrier dependent or respiratory compromise due to myopathy

Pyramidal

- ◯ Normal
- ◯ Mild. Unilateral pyramidal signs but retaining functional movement
- ◯ Moderate. Dense hemiplegia with little movement of affected side
- ◯ Severe. Bilateral pyramidal weakness with little or no movement

Extrapyramidal
- ◯ Normal
- ◯ MIld. Unilateral extrapyramidal posturin and increased tone
- ◯ Moderate. Bilateral extrapyramidal posturing and increased tone
- ◯ Severe. Severe extrapyramidal posturing resulting in very little movement

Neuropathy
- ◯ Normal
- ◯ Mild. Areflexia only
- ◯ Moderate. Sensory ataxia or motor impairment (distal weakness) but mobile
- ◯ Severe. REliant on mobility aids primarily due to neuropathy

Section III score ________________________________

Section IV: Quality of life

This survey asks for your views about your child's recent health. Please answer every question by marking an "x" in the box next to the phrase which best describes your answer

1. During the past 4 weeks, how would you rate your child's overall health?
◯ Very poor ◯ Poor ◯ Fair ◯ Good ◯ Very good

2. During the past 4 weeks, how much did your child's physical health problems limit their physical activities (such as moving or playnig)
◯ Very much ◯ Quite a lot ◯ Somewhat ◯ A little ◯ Not at all

3. During the past 4 weeks, how much energy did your child have?
◯ None ◯ A little ◯ Some ◯ Quite a lot ◯ Very much

4. During the past 4 weeks, how much bodily pain/ discomfort did your child have
◯ Very much ◯ quite a lot ◯ Some ◯ A little ◯ None

5. During the past 4 weeks, how would you rate your child's behaviour compared with other children his/her age?
◯ Very poor ◯ Poor ◯ Fair ◯ Good ◯ Very good

6. During the past 4 weeks, how would you rate your child's ability to interact with other people (e.g. with you, siblings or other children his/her age) compared with other children his/her age?
◯ Very poor ◯ Poor ◯ Fair ◯ Good ◯ Very good

Total score ________________________________

THE NEWCASTLE MITOCHONDRIAL DISEASE ADULT SCALE (NMDAS)

[Attachment: "The newcastle mitochondrial disease adult scale (NMDAS).pdf"]

NMDAS date ________________________________

Section I: Current function (preceding 4 week period)

Vision

- ◯ Normal
- ◯ No functional impairment but aware of worsened acuities
- ◯ Mild- difficulty with small print or text on television
- ◯ Moderate- difficulty outside the home (eg buns numbers, road signs or shopping)
- ◯ Severe- difficulty recognisisng faces
- ◯ Unable to navigate without help (eg carer, dog, cane)

Hearing with our without hearing aid

- ◯ Normal
- ◯ No communication problems but aware of tinnitus or derioration from prior "normal" hearing.
- ◯ Mild deafness (eg missing words in presence of background noise). Fully corrected with hearing aid.
- ◯ Moderate deafness (eg regularly requiring repetition). Not fully corrected with hearing aid.
- ◯ Severe deafness- poor hearing even with aid (see 3 above)
- ◯ End stage-virtually no hearing despite aid. Relies heavily on non-verbal communication (eg lip reading) or has cochlear implant.

Speech

- ◯ Normal
- ◯ Communication unaffected but patient or others awarre of changes in speech patterns or quality
- ◯ Mild difficulties- usually understood and rarely asked to repeat things
- ◯ Moderate difficulties- poorly understood by strangers and frequently asked to repeat things
- ◯ Severe difficulties- poorly undestood by family or friends.
- ◯ Not understood by family or friends. Rquires communication aid.

Swallowing

- ◯ Normal
- ◯ Mild- sensation of solids "sticking" (occasional)
- ◯ Sensation of solids "Sticking" (most meals) or need to modify diet (eg avoidance of steak/ sald).
- ◯ Difficulty swallowing solids- a ffecting meal size or duration. Coughing, choking or nasal regurgitation infrequent (1 to 4 times per month) but more than peers.
- ◯ Requires adapted diet- regular coughing, choking, or nasal regurgitation (more than once per week).
- ◯ Rquiring enteral feeding (eg. PEG)

Handwriting

- ◯ Normal
- ◯ Writting speed unaffected but aware of increasing untidiness
- ◯ Mild- Has to write slower to maintain tidiness/legibility
- ◯ Moderate- Handwriting takes at least twice as long or resorts to printing (must previously have used joined writing)
- ◯ Sever. Handwriting mostly ilegible. Printing very slow and untidiy
- ◯ Unable towrite. No legible words

Cutting food and handling utensils

- ◯ Normal
- ◯ Slightly slow and/or clumsy but minimal effect on meal duration.
- ◯ Slow and/or clumsy with extended meal duration, but no help required
- ◯ Difficulty cutting up food and inaccuracy of transfer pronounced. Can manage alone but avoids problem foods (eg peas) or carer typically offers minor assistance (eg cutting up steak).
- ◯ Unable to cut up food. Can pass food to mouth with great effort or inaccuracy. Resultant intake minimal. Requires major assistance
- ◯ Needs to be fed

Dressing

- ◯ Normal
- ◯ Occasional difficulties (eg shoe laces, buttons etc) but no real impact on time or effort taken to dress.
- ◯ Mild - Dressing takes longer and requires more effort than expected at the patient's age. No help required.
- ◯ Moderate - Can dress unaided but takes at least twice as long and is a major effort. Carer typically helps with difficult tasks such as shoe laces or buttons
- ◯ Severe - Unable to dress without help but some tasks completed unaided
- ◯ Needs to be dressed

Hygiene

- ◯ Normal
- ◯ Occasional difficulties only but no real impact on time or effort required.
- ◯ Mild - hygienic care takes longer but quality unaffected
- ◯ Moderate - bathes and showers alone with difficulty or needs bath chair / modifications. Dextrous tasks (eg brushing teeth, combing hair) performed poorly.
- ◯ Severe - unable to bathe or shower without help. Major difficulty using toilet alone. Dextrous task require help.
- ◯ Dependent upon carers to wash, bathe, and toilet

Exercise Tolerance

- ◯ Normal
- ◯ Unlimited on flat - symptomatic on inclines or stairs
- ◯ Able to walk < 1000m on the flat. Restricted on inclines or stairs - rest needed after 1 flight (12 steps).
- ◯ Able to walk < 500m on the flat. Rest needed after 8 steps on stairs.
- ◯ Able to walk < 100m on the flat. Rest needed after 4 steps on stairs.
- ◯ Able to walk < 25m on the flat. Unable to do stairs alone.

Gait stability

- ◯ Normal.
- ◯ Normal gait - occasional difficulties on turns, uneven ground, or if required to balance on narrow base.
- ◯ Gait reasonably steady. Aware of impaired balance. Occasionally off balance when walking.
- ◯ Unsteady gait. Always off balance when walking. Occasional falls. Gait steady with support of stick 6, or person.
- ◯ Gait grossly unsteady without support. High likelihood of falls. Can only walk short distances (< 10m) 8, without support.
- ◯ Unable to walk without support. Falls on standing.

Section II. System Specific Involvement (preceding 12 month period)

Psychiatric

- ◯ None
- ◯ Mild & transient (eg reactive depression) - lasting less than 3 months
- ◯ Mild & persistent (lasting more than 3 months) or recurrent. Patient has consulted GP
- ◯ Moderate & warranting specialist treatment (e.g. from a psychiatrist) - eg. bipolar disorder or depression with vegetative symptoms (insomnia, anorexia, abulia etc).
- ◯ Severe (eg self harm - psychosis etc).
- ◯ Institutionalised or suicide attempt.

Migraine Headaches during the last 3 months (how many days have headaches prevented the patient from funcitoning normally at school, work or in the home?

- ◯ No past history
- ◯ Asymptomatic but past history of migraines
- ◯ One day per month
- ◯ Two days per month
- ◯ Three days per month
- ◯ Four days per month or more

Seizures

- ◯ No past history
- ◯ Asymptomatic but past history of epilepsy
- ◯ Myoclonic or simple partial seizures only
- ◯ Multiple absence, complex partial, or myoclonic seizures affecting function or single generalised seizure
- ◯ Multiple generalised seizures.
- ◯ Status epilepticus

Stroke-like-episodes (exclude focal deficits felt to be of vascular aetiology)	◯ None<br>◯ Transient focal sensory symptoms only (less than 24 hours).<br>◯ Transient focal motor symptoms only (less than 24 hours).<br>◯ Single stroke-like episode affecting one hemisphere (more than 24 hours).<br>◯ Single stroke-like episode affecting both hemispheres (more than 24 hours).<br>◯ Multiple stroke-like episodes (more than 24 hours each).
Encephalopathic episodes	◯ No past history<br>◯ Asymptomatic but past history of encephalopathy.<br>◯ Mild - single episode of personality or behavioural change but retaining orientation in time/place/person<br>◯ Moderate - single episode of confusion or disorientation in time, place or person<br>◯ Severe - multiple moderate episodes (as above) or emergency hospital admission due to encephalopathy without associated seizures or stroke-like episodes.<br>◯ Very severe - in association with seizures, strokes or gross lactic acidaemia
Gastro-intestinal symptoms	◯ None<br>◯ Mild constipation only or past history of bowel resection for dysmotility<br>◯ Occasional symptoms of 'irritable bowel' (pain, bloating or diarrhoea) with long spells of normality.<br>◯ Frequent symptoms (as above) most weeks or severe constipation with bowels open less than once/week or need for daily medications.<br>◯ Dysmotility requiring admission or persistent and/or recurrent anorexia/vomiting/weight loss.<br>◯ Surgical procedures or resections for gastrointestinal dysmotility
Diabetes mellitus	◯ None<br>◯ Past history of gestational diabetes or transient glucose intolerance related to intercurrent illness.<br>◯ Impaired glucose tolerance (in absence of intercurrent illness).<br>◯ NIDDM (diet).<br>◯ NIDDM (tablets).<br>◯ DM requiring insulin (irrespective of treatment at onset).
Respiratory muscle weakness	◯ FVC normal (≥ 85% predicted).<br>◯ FVC < 85% predicted.<br>◯ FVC < 75% predicted.<br>◯ FVC < 65% predicted.<br>◯ FVC < 55% predicted.<br>◯ FVC < 45% predicted or ventilatory support for over 6 hours per 24 hr period (not for OSA alone).

Cardiovascular system

◯ None
◯ Asymptomatic ECG change
◯ Asymptomatic LVH on echo or non-sustained brady/tachyarrhythmia on ECG
◯ Sustained or symptomatic arrhythmia, LVH or cardiomyopathy. Dilated chambers or reduced function on echo. Mobitz II AV block or greater.
◯ Requires pacemaker, defibrillator, arrhythmia ablation, or LVEF < 35% on echocardiogram.
◯ Symptoms of left ventricular failure with clinical and/or x-ray evidence of pulmonary oedema or LVEF < 30% on echocardiogram.

Section III. Current Clinical Assessment (at the time of assessment)

Visual acuity (with usual glasses, contact lenses or pinhole)

◯ CSD ≤ 12 (ie normal vision - 6/6, 6/6 or better).
◯ CSD ≤ 18 (eg 6/9, 6/9).
◯ CSD ≤ 36 (eg 6/12, 6/24).
◯ CSD ≤ 60 (eg 6/24, 6/36).
◯ CSD ≤ 96 (eg 6/60, 6/36).
◯ CSD ≥ 120 (eg 6/60, 6/60 or worse).

Ptosis

◯ None
◯ Mild ptosis - not obscuring either pupil.
◯ Unilateral ptosis obscuring < 1/3 of pupil.
◯ Bilateral ptosis obscuring < 1/3 or unilateral ptosis obscuring > 1/3 of pupil or prior unilateral surgery
◯ Bilateral ptosis obscuring > 1/3 of pupils or prior bilateral surgery
◯ Bilateral ptosis obscuring >2/3 of pupils or >1/3 of pupils despite prior bilateral surgery.

Chronic Progressive External Ophthalmoplegia

◯ None
◯ Some restriction of eye movement (any direction). Abduction complete.
◯ Abduction of worst eye incomplete.
◯ Abduction of worst eye below 60% of normal
◯ Abduction of worst eye below 30% of norma
◯ Abduction of worst eye minimal (flicker)

Dysphonia/Dysarthria

◯ None.
◯ Minimal - noted on examination only.
◯ Mild - clear impairment but easily understood.
◯ Moderate - some words poorly understood and infrequent repetition needed.
◯ Severe - many words poorly understood and frequent repetition needed.
◯ Not understood. Requires communication aid.

Myopathy

- ◯ Normal.
- ◯ Minimal reduction in hip flexion and/or shoulder abduction only (eg MRC 4+/5).
- ◯ Mild but clear proximal weakness in hip flexion and shoulder abduction (MRC 4/5). Minimal weakness in elbow flexion and knee extension (MRC 4+/5 - both examined with joint at 90 degrees).
- ◯ Moderate proximal weakness including elbow flexion & knee extension (MRC 4/5 or 4 -/5) or difficulty rising from a 90 degree squat.
- ◯ Waddling gait. Unable to rise from a 90 degree squat (=a chair) unaided.
- ◯ Wheelchair dependent primarily due to proximal weakness.

Cerebellar ataxia

- ◯ None.
- ◯ Normal gait but hesitant heel-toe.
- ◯ Gait reasonably steady. Unable to maintain heel-toe walking or mild UL dysmetria.
- ◯ Ataxic gait (but walks unaided) or UL intention tremor & past-pointing. Unable to walk heel-toe - falls immediately.
- ◯ Severe - gait grossly unsteady without support or UL ataxia sufficient to affect feeding.
- ◯ Wheelchair dependent primarily due to ataxia or UL ataxia prevents feeding.

Neuropathy

- ◯ None.
- ◯ Subtle sensory symptoms or areflexia.
- ◯ Sensory impairment only (eg glove & stocking sensory loss).
- ◯ Motor impairment (distal weakness) or sensory ataxia.
- ◯ Sensory ataxia or motor effects severely limit ambulation.
- ◯ Wheelchair bound primarily due to sensory ataxia or neurogenic weakness

Pyramidal Involvement

- ◯ None.
- ◯ Focal or generalised increase in tone or reflexes only.
- ◯ Mild focal weakness, sensory loss or fine motor impairment (eg cortical hand).
- ◯ Moderate hemiplegia allowing unaided ambulation or dense UL monoplegia.
- ◯ Severe hemiplegia allowing ambulation with aids or moderate tetraplegia (ambulant).
- ◯ Wheelchair dependant primarily due to hemiplegia or tetraplegia.

Extrapyramidal

- ◯ Normal.
- ◯ Mild and unilateral. Not disabling (H&Y stage 1).
- ◯ Mild and bilateral. Minimal disability. Gait affected (H&Y stage 2).
- ◯ Moderate. Significant slowing of body movements (H&Y stage 3)
- ◯ Severe. Rigidity and bradykinesia. Unable to live alone. Can walk to limited extent (H&Y stage 4).
- ◯ Cannot walk or stand unaided. Requires constant nursing care (H&Y stage 5).

Cognition (patient undergo testing using WTAR, Symbol Search and Speed of Comprehension Test

◯ Combined centiles 100 or more.
◯ Combined centiles 60 - 99
◯ Combined centiles 30 - 59
◯ Combined centiles 15 - 29
◯ Combined centiles 5 - 14
◯ Combined centiles 4 or below.

THE NEWCASTLE PAEDIATRIC MITOCHONDRIAL DISEASE SCALE (NPMDS)

[Attachment: "NPMDS escala 2 - 11 años-1.pdf"]

NPMDS date ________________________________

Section I: Current Function (preceding 4 week period)

Vision

◯ Normal. No parental / patient concerns
◯ Mild. Inattention to small objects in visual field or parent concerned about abnormality of visual behaviour
◯ Moderate. Visual impairment not fully corrected with glasses or inattention to large objects in visual field
◯ Severe. Not recognising faces or registered blind or using additional visual aids

Hearing

◯ Normal
◯ Mild. Requires regular repetition / raised voice or not reacting to loud sounds
◯ Moderate. Hearing impaired but fully corrected with hearing aid
◯ Severe. Poor hearing even with aid

Communication

◯ Normal. Age appropriate communication
◯ Mild. Verbal communication impaired. Supplemented by alternative methods (e.g. signing, pointing)
◯ Moderate. Not communicating effectively with strangers (irrespective of methods)
◯ Severe. Not communicating effectively with parents (irrespective of methods)

Feeding

◯ Normal
◯ Mild. Choking / vomiting / anorexia resulting in reduced intake or adaptation of age appropriate diet
◯ Moderate. Supplementary enteral feeding or recurrent aspiration pneumonia
◯ Severe. Exclusive enteral feeding (gastrostomy / NG tube). Nil by mouth

Self-care (personal hygiene, dressing, utensil use)

◯ Normal. No concerns. Age appropriately reliant on carers / parents
◯ Mild. Requires help with some age appropriate tasks
◯ Moderate. Requires help with all age appropriate tasks
◯ Severe. Reliant on parents with no contribution to self care

Mobility

◯ Normal. No concerns. Age appropriate mobility
◯ Mild. Difficulty walking up stairs or inclines
◯ Moderate. Requires support (stick / frame / callipers) to walk on the flat
◯ Severe. Wheelchair / carrier dependent

Educational achievement

◯ Normal. Academic achievement comparable to peers in mainstream school / nursery
◯ Mild. Struggling to remain in mainstream school / nursery
◯ Moderate. Attending special school / nursery
◯ Severe. Not attending school / nursery primarily due to illness

Section II: System Specific Involvement (preceding 6 month period)

Seizures

◯ None
◯ Mild. Myoclonic or absence seizures only or < 1 generalised tonic-clonic seizure/month
◯ Moderate. > 5 generalized tonic-clonic seizures/month or > 20 absence or myoclonic seizures/month
◯ Severe. Status epilepticus

Encephalopathy

◯ None
◯ Mild. Single episode of personality change, excessive sleepiness, confusion or disorientation
◯ Moderate. Obtunded or >2 encephalopathic episodes/year
◯ Severe. Life-threatening encephalopathy- requires artificial ventilation

Stroke-like Episodes

◯ None
◯ Mild. Transient motor symptoms lasting < 24 hours
◯ Moderate. Single stroke-like episode (> 24 hours)
◯ Severe. Multiple stroke-like episodes (> 24 hours each)

Gastrointestinal

◯ Normal
◯ Mild. Mild constipation or unexplained vomiting / diarrhoea < 1/week
◯ Moderate. Moderate constipation (some relief with laxative treatment) or unexplained vomiting / diarrhoea > 3/week
◯ Severe. Severe constipation (no relief with laxative treatment) or unexplained vomiting / diarrhoea every day or surgical intervention for dysmotility

Endocrine

◯ Normal
◯ Mild. Biochemical evidence of impaired function
◯ Moderate. Endocrine failure requiring replacement therapy
◯ Severe. Decompensation (e.g. diabetic ketoacidosis, Addinsonian crisis)

Respiratory

- ◯ Normal
- ◯ Mild. Abnormal respiration not requiring hospitalisation
- ◯ Moderate. Abnormal respiration requiring hospitalisation but not ventilation
- ◯ Severe. Abnormal respiration requiring artificial ventilation

Cardiovascular (over preceding 12 months)

- ◯ Normal
- ◯ Mild. Asymptomatic ECG change
- ◯ Moderate. Abnormal echocardiogram (e.g. cardiomegaly) or sustained / symptomatic arrhythmia on ECG
- ◯ Severe. Decompensated cardiomyopathy or requiring pacing device / defibrillator / ablation

Renal

- ◯ Normal
- ◯ Mild. Impaired function but no change in diet or therapy required
- ◯ Moderate. Impaired function requiring restricted protein diet
- ◯ Severe. Failure requiring transplant / dialysis

Liver

- ◯ Normal
- ◯ Mild. Impaired function but no change in diet or therapy required
- ◯ Moderate. Impaired function requiring restricted protein diet
- ◯ Severe. Failure requiring transplant / dialysis

Blood

- ◯ Normal
- ◯ Mild. Anaemia only
- ◯ Moderate. Asymptomatic pancytopenia
- ◯ Severe. Pancytopenia requiring regular transfusion / transplantation

---

Section III: Current Clinical Assessment (at the time of assessment)

Growth (ht and Weight) over preceding 6 months

- ◯ Normal. Following normal growth trajectory
- ◯ Mild. Height or weight or both less than 2nd centile but growing parallel to it
- ◯ Moderate. Height or weight or both crossing one centile
- ◯ Severe. Height or weight or both crossing ≥ 2 centiles or less than 2nd centile with divergent trajectory

Development over preceding 6 months Score: ________________________________

Vision (with usual glasses in the better eye)

- ◯ Normal. Visual acuity better than or equal to 6/12 or normal fixation and tracking
- ◯ Mild. Acuity worse than 6/12 but better than or equal to 6/18 or no fixation on small objects
- ◯ Moderate. Acuity worse than 6/18 but better than or equal to 6/60 or impaired fixation on large, brightly coloured objects
- ◯ Severe. Acuity worse than 6/60 or no response to light or visual threat or unable to finger count

Ptosis and eye movement

- ◯ Normal
- ◯ Mild. Gaze evoked nystagmus or unilateral ptosis or impaired eye movement at extremities
- ◯ Moderate. Intermittent nystagmus at rest or bilateral ptosis not obscuring pupils or restriction of >50% eye movement
- ◯ Severe. Continuous nystagmus at rest or bilateral ptosis obscuring pupils or only a flicker of eye movement

Myopathy

- ◯ Normal
- ◯ Mild. Mild symmetrical weakness of hip and / or shoulder girdle only
- ◯ Moderate. Moderate symmetrical weakness (proximal>distal) limiting mobility
- ◯ Severe. Wheelchair / carrier dependent or respiratory compromise due to myopathy

Ataxia

- ◯ Normal
- ◯ Mild. Ataxic gait but walks unaided or mild upper limb dysmetria
- ◯ Moderate. Gait abnormality requiring assistance or severe upper limb dysmetria
- ◯ Severe. Wheelchair dependent or unable to feed due to ataxia

Pyramidal

- ◯ Normal
- ◯ Mild. Focal dystonia or unilateral extrapyramidal tremor / bradykinesia
- ◯ Moderate. Generalised dystonia or bilateral extrapyramidal tremor / bradykinesia
- ◯ Severe. Wheelchair dependent due to extrapyramidal disorder

Neuropathy

- ◯ Normal.
- ◯ Mild. Areflexia only
- ◯ Moderate. Sensory ataxia or motor impairment (distal weakness) but mobile
- ◯ Severe. Reliant on mobility aids primarily due to neuropathy

---

Section IV: Quality of Life-Parent Completed

During the past 4 weeks, how would you rate your child's overall health?

◯ Very poor ◯ Poor ◯ Fair ◯ Good ◯ Very good

During the past 4 weeks, how much did your child's physical health problems limit their physical activities (such as walking or playing with friends)?

◯ Very much ◯ Quite a lot ◯ Some what ◯ A little ◯ Not at all

During the past 4 weeks, how much did your child's health problems limit their everyday life (such as attending school, caring for themselves) compared with a person of the same age in perfect health?

◯ Very much ◯ Quite a lot ◯ Somewhat ◯ A little ◯ Not at all

During the past 4 weeks, how much energy did your child have?

◯ None ◯ A little ◯ Some ◯ Quite a lot ◯ Very much

During the past 4 weeks, how much bodily pain/discomfort did your child have?	◯ Very much ◯ Quite a lot ◯ Some ◯ A little ◯ None
During the past 4 weeks, how much was your child bothered by emotional problems?	◯ Very much ◯ Quite a lot ◯ Somewhat ◯ A little ◯ Not at all
During the past 4 weeks, how satisfied did your child feel with their abilities, looks, relationships with other people and life in general?	◯ Very dissatisfied ◯ Quite dissatisfied ◯ Neither dissatisfied or satisfied ◯ Quite satisfied ◯ Very satisfied
During the past 4 weeks, how would you rate your child's behaviour compared with other children his / her age?	◯ Very poor ◯ Poor ◯ Fair ◯ Good ◯ Very good
During the past 4 weeks, how would you rate your child's ability to interact with other people (e.g. making friends, talking to other children / strangers) compared with other children his / her age?	◯ Very poor ◯ Poor ◯ Fair ◯ Good ◯ Very good
During the past 4 weeks, how much were you (the parent / carer) bothered by emotional problems (e.g. feelings of anxiety, sadness) as a result of your child's illness?	◯ Very ◯ Quite a lot ◯ Somewhat ◯ A little ◯ Not at all
During the past 4 weeks, how much was your time limited as a result of your child's illness?	◯ Very ◯ Quite a lot ◯ Somewhat ◯ A little ◯ Not at all
During the past 4 weeks, how much were your family's activities limited or interrupted as a result of your child's illness?	◯ Very ◯ Quite a lot ◯ Somewhat ◯ A little ◯ Not at all
During the past 6 months, what has been the financial cost of your child's illness?	◯ Very expensive ◯ Quite expensive ◯ Moderately expensive ◯ Little additional cost ◯ No additional cost
During the past 4 weeks, how would you rate your family's ability to get along with one another?	◯ Very poor ◯ Poor ◯ Fair ◯ Good ◯ Very good
During the past 4 weeks, how often did your child's illness have a positive effect on your child, you or your family (e.g. being treated well due to illness, meeting new people)?	◯ Never ◯ Occasionally ◯ Sometimes ◯ Quite a lot ◯ Most of the time

Section IV: Quality of Life-Self Completed (7-11 years)

During the past 4 weeks, how would you rate your overall health?	◯ Very poor ◯ Poor ◯ Fair ◯ Good ◯ Very good
During the past 4 weeks, how much did your physical health problems limit your physical activities (such as walking or playing with friends)?	◯ Very much ◯ Quite a lot ◯ Somewhat ◯ A little ◯ Not at all
During the past 4 weeks, how much did your health problems limit your everyday life (such as going to school, caring for yourself) compared with a person of your age in perfect health?	◯ Very much ◯ Quite a lot ◯ Somewhat ◯ A little ◯ Not at all

During the past 4 weeks, how much energy did you have?

○ None ○ A little ○ Some
○ Quite a lot ○ Very much

During the past 4 weeks, how much pain or discomfort did you have?

○ Very much ○ Quite a lot
○ Some ○ A little ○ None

During the past 4 weeks, how much were you bothered by emotional problems (such as feeling sad or frightened)?

○ Very much ○ Quite a lot
○ Somewhat ○ A little
○ Not at all

During the past 4 weeks, how satisfied did you feel with your abilities, looks, relationships with other people and life in general?

○ Very dissatisfied ○ Quite dissatisfied
○ Nither satisfied or dissatisfied
○ Quite satisfied ○ Very satisfied

During the past 4 weeks, how would you rate your behaviour compared with other children your age?

○ Very poor ○ Poor ○ Fair
○ Good ○ Very good

During the past 4 weeks, how easy did you find it to get on with other people (e.g. making friends, talking to other children / strangers) compared with other children your age?

○ Very hard ○ Hard
○ Ok ○ Easy ○ Very easy

During the past 4 weeks, how would you rate your family's ability to get along with one another?

○ Very poor ○ Poor ○ Fair
○ Good ○ Very good

During the past 4 weeks, how often did your illness have a positive effect on you or your family (e.g. being treated well because of your illness, meeting new people)?

○ Never ○ Occasionally
○ Sometimes ○ Quite a lot
○ Most of the time

THE NEWCASTLE PAEDIATRIC MITOCHONDRIAL DISEASE RATINGS SCALES (NPMDS)

[Attachment: "NPMDS escala 12 - 18 años-1.pdf"]

NPMDS date ________________________________

Section I: Function (preceding 4 week period)

Vision (with usual glasses)

○ Normal. No parental / patient concerns
○ Mild. Difficulty with small print or text on the television
○ Moderate. Difficulty outside the home (e.g. bus numbers, road signs or shopping)
○ Severe. Difficulty recognising faces or unable to navigate without help or registered blind

Hearing

○ Normal
○ Mild. Mild deafness (e.g. missing words in presence of background noise)
○ Moderate. Hearing impaired but fully corrected with hearing aid
○ Severe. Poor hearing even with aid or reliant on non-verbal communication

Communication

○ Normal
○ Mild. Mild speech difficulties but easily understood and rarely asked to repeat things
○ Moderate. Speech poorly understood by strangers and frequently asked to repeat things
○ Severe. Speech not understood by family or friends and / or requires communication aid

Feeding

◯ Normal
◯ Mild. Choking / vomiting / anorexia resulting in reduced intake or swallowing difficulties requiring dietary adaptations
◯ Moderate. Supplementary enteral feeding or recurrent aspiration pneumonia
◯ Severe. Exclusive enteral feeding (gastrostomy / NG tube). Nil by mouth

Self-care (personal hygiene, dressing, utensil use)

◯ Normal
◯ Mild. Slow and / or clumsy self-care requiring extra time or effort. Requires help with some tasks
◯ Moderate. Requires help with most tasks but retains some independent self-care function
◯ Severe. Requires one to one assistance

Mobility

◯ Normal
◯ Mild. Difficulty walking up stairs or inclines
◯ Moderate. Requires support (stick / frame / callipers) to walk on the flat
◯ Severe. Wheelchair dependent

Educational achievement

◯ Normal. Academic achievement comparable to peers in mainstream school
◯ Mild. Struggling to remain in mainstream school
◯ Moderate. Attending special school
◯ Severe. Not attending school primarily due to illness

Section I score ______________________________

Section II: System Specific Involvement (preceding 12 month period)

Seizures

◯ None
◯ Mild. Myoclonic or absence seizures only or < 1 generalised tonic-clonic seizure each month
◯ Moderate. > 5 generalized tonic-clonic seizures/month or > 20 absence or myoclonic seizures/month
◯ Severe. Status epilepticus

Encephalopathy

◯ None
◯ Mild. Single episode of personality change, excessive sleepiness, confusion or disorientation
◯ Moderate. Obtunded or >2 encephalopathic episodes/year
◯ Severe. Life-threatening encephalopathy- requires artificial ventilation

Stroke-like episodes

◯ None
◯ Mild. Transient motor symptoms lasting < 24 hours
◯ Moderate. Single stroke-like episode (> 24 hours)
◯ Severe. Multiple stroke-like episodes (> 24 hours each)

Gastrointestinal

- ◯ Normal
- ◯ Mild. Mild constipation or unexplained vomiting / diarrhoea < 1/week
- ◯ Moderate. Moderate constipation (some relief with laxative treatment) or unexplained vomiting / diarrhoea > 3/week
- ◯ Severe. Severe constipation (no relief with laxative treatment) or unexplained vomiting / diarrhoea every day or surgical intervention for dysmotility

Endocrine

- ◯ Normal
- ◯ Mild. Biochemical evidence of impaired function
- ◯ Moderate. Endocrine failure requiring replacement therapy
- ◯ Severe. Endocrine decompensation (e.g. diabetic ketoacidosis, Addisonian crisis)

Respiratory

- ◯ Normal
- ◯ Mild. Abnormal respiration not requiring hospitalisation
- ◯ Moderate. Abnormal respiration requiring hospitalisation but not ventilation
- ◯ Severe. Abnormal respiration requiring artificial ventilation

Cardiovascular

- ◯ Normal
- ◯ Mild. Asymptomatic ECG change
- ◯ Moderate. Abnormal echocardiogram (e.g. cardiomegaly) or sustained / symptomatic arrhythmia on ECG
- ◯ Severe. Decompensated cardiomyopathy or requiring pacing device / defibrillator / ablation

Renal

- ◯ Normal
- ◯ Mild. Impaired function but no change in diet or therapy required
- ◯ Moderate. Impaired function requiring restricted protein diet
- ◯ Severe. Failure requiring transplant / dialysis

Liver

- ◯ Normal
- ◯ Mild. Mildly impaired Liver Function Tests (LFTs). No symptoms of hepatic failure
- ◯ Moderate. Impaired LTFs with symptoms (e.g. jaundice, oedema)
- ◯ Severe. Failure requiring hospitalisation and / or transplantation

Blood

- ◯ Normal
- ◯ Mild. Anaemia only
- ◯ Moderate. Asymptomatic pancytopenia
- ◯ Severe. Pancytopenia requiring regular transfusion / transplantation

Section II total score ________________________________

Section III: Current Clinical Assessement (at the time of the assessesment unless otherwise stated in the question)

Growth (ht and weight) over preceding 6 months

- ◯ Normal. Following normal growth trajectory
- ◯ Mild. Height or weight or both less than 2nd centile but growing parallel to it
- ◯ Moderate. Height or weight or both crossing one centile
- ◯ Severe. Height or weight or both crossing ≥ 2 centiles or less than 2nd centile with divergent trajectory

Development over preceding 12 months score ________________________________

Vision (with usual glasses, better eye)

- ◯ Normal. Following normal growth trajectory
- ◯ Mild. Height or weight or both less than 2nd centile but growing parallel to it
- ◯ Moderate. Height or weight or both crossing one centile
- ◯ Severe. Height or weight or both crossing ≥ 2 centiles or less than 2nd centile with divergent trajectory

Ptosis and eye movement

- ◯ Normal
- ◯ Mild. Gaze evoked nystagmus or unilateral ptosis or impaired eye movement at extremities
- ◯ Moderate. Intermittent nystagmus at rest or bilateral ptosis not obscuring pupils or restriction of >50% eye movement
- ◯ Severe. Continuous nystagmus at rest or bilateral ptosis obscuring pupils or only a flicker of eye movement

Myopathy

- ◯ Normal
- ◯ Mild. Mild symmetrical weakness of hip and / or shoulder girdle only
- ◯ Moderate. Moderate symmetrical weakness (proximal > distal) limiting mobility
- ◯ Severe. Wheelchair dependent or respiratory compromise due to myopathy

Ataxia

- ◯ Normal
- ◯ Mild. Ataxic gait but walks unaided or mild upper limb dysmetria
- ◯ Moderate. Gait abnormality requiring assistance or severe upper limb dysmetria
- ◯ Severe. Wheelchair dependent or unable to feed due to ataxia

Pyramidal

- ◯ Normal
- ◯ Mild. Mild hemiplegia allowing unaided ambulation
- ◯ Moderate. Moderate hemiplegia allowing ambulation with aids
- ◯ Severe. Wheelchair dependent due to hemi / tetraplegia

Extrapyramidal
- ◯ Normal
- ◯ Mild. Focal dystonia or unilateral extrapyramidal tremor / bradykinesia
- ◯ Moderate. Generalised dystonia or bilateral extrapyramidal tremor / bradykinesia
- ◯ Severe. Wheelchair dependent due to extrapyramidal disorder

Neuropathy
- ◯ Normal.
- ◯ Mild. Areflexia only
- ◯ Moderate. Sensory ataxia or motor impairment (distal weakness) but mobile
- ◯ Severe. Reliant on mobility aids primarily due to neuropathy

Section III score ________________________________

Section IV: Quality of life-Parent Completed

During the past 4 weeks, how would you rate your child's overall health?
◯ Very poor ◯ Poor ◯ Fair ◯ Good ◯ Very good

During the past 4 weeks, how much did your child's physical health problems limit their physical activities (such as walking or playing with friends)?
◯ Very much ◯ Quite a lot ◯ Somewhat ◯ A little ◯ Not at all

During the past 4 weeks, how much did your child's health problems limit their everyday life (such as attending school, caring for themselves) compared with a person of the same age in perfect health?
◯ Very much ◯ Quite a lot ◯ Somewhat ◯ A little ◯ Not at all

During the past 4 weeks, how much energy did your child have?
◯ None ◯ A little ◯ Some ◯ Quite a lot ◯ Very much

During the past 4 weeks, how much bodily pain/discomfort did your child have?
◯ Very much ◯ Quite a lot ◯ Some ◯ A little ◯ None

During the past 4 weeks, how much was your child bothered by emotional problems?
◯ Very much ◯ Quite a lot ◯ Somewhat ◯ A little ◯ Not at all

During the past 4 weeks, how satisfied did your child feel with their abilities, looks, relationships with other people and life in general?
◯ Very dissatisfied ◯ Quite dissatisfied ◯ Neither dissatisfied or satisfied ◯ Quite satisfied ◯ Very satisfied

During the past 4 weeks, how would you rate your child's behaviour compared with other children his / her age?
◯ Very poor ◯ Poor ◯ Fair ◯ Good ◯ Very good

During the past 4 weeks, how would you rate your child's ability to interact with other people (e.g. making friends, talking to other children / strangers) compared with other children his / her age?
◯ Very poor ◯ Poor ◯ Fair ◯ Good ◯ Very good

During the past 4 weeks, how much were you (the parent / carer) bothered by emotional problems (e.g. feelings of anxiety, sadness) as a result of your child's illness?
◯ Very ◯ Quite a lot ◯ Somewhat ◯ A little ◯ Not at all

During the past 4 weeks, how much was your time limited as a result of your child's illness?
◯ Very ◯ Quite a lot
◯ Somwhat ◯ A littel
◯ Not at all

During the past 4 weeks, how much were your family's activities limited or interrupted as a result of your child's illness?
◯ Very ◯ Quite a lot
◯ Somwhat ◯ A little
◯ Not at all

During the past 6 months, what has been the financial cost of your child's illness?
◯ Very expensive ◯ Quite expensive
◯ Moderately expensive
◯ Little additional cost
◯ No additional cost

During the past 4 weeks, how would you rate your family's ability to get along with one another?
◯ Very poor ◯ Poor ◯ Fair
◯ Good ◯ Very good

During the past 4 weeks, how often did your child's illness have a positive effect on your child, you or your family (e.g. being treated well due to illness, meeting new people)?
◯ Never ◯ Occasionally
◯ Sometimes ◯ Quite a lot
◯ Most of the time

Section D: Quality of life-Self completed

During the past 4 weeks, how would you rate your overall health?
◯ Very poor ◯ Poor ◯ Fair
◯ Good ◯ Very good

During the past 4 weeks, how much did your physical health problems limit your physical activities (such as walking or going out with friends)?
◯ Very much ◯ Quite a lot
◯ Somewhat ◯ A little
◯ Not at all

During the past 4 weeks, how much did your health problems limit your everyday life (such as going to school, caring for yourself) compared with a person of your age in perfect health?
◯ Very much ◯ Quite a lot
◯ Somewhat ◯ A little
◯ Not at all

During the past 4 weeks, how much energy did you have?
◯ None ◯ A little ◯ Some
◯ Quite a lot ◯ Very much

During the past 4 weeks, how much pain or discomfort did you have?
◯ Very much ◯ Quite a lot
◯ Some ◯ A little ◯ None

During the past 4 weeks, how much were you bothered by emotional problems (e.g. feeling sad or frightened)?
◯ Very much ◯ Quite a lot
◯ Somewhat ◯ A little
◯ Not at all

During the past 4 weeks, how satisfied did you feel with their abilities, looks, relationships with other people and life in general?
◯ Very dissatisfied ◯ Quite dissatisfied
◯ Neither dissatisfied or satisfied
◯ Quite satisfied ◯ Very satisfied

During the past 4 weeks, how would you rate your behaviour compared with people your age?
◯ Very poor ◯ Poor ◯ Fair
◯ Good ◯ Very good

During the past 4 weeks, how easy did you find it to get on with other people (e.g. making friends, talking to friends / strangers) compared with other people your age?
◯ Very hard ◯ Hard
◯ Fair ◯ Good ◯ Very good

During the past 4 weeks, how would you rate your family's ability to get along with one another?

◯ Very poor ◯ Poor ◯ Fair
◯ Good ◯ Very good

During the past 4 weeks, how often did your illness have a positive effect on you or your family (e.g. being treated well because of your illness, meeting new people)?

◯ Never ◯ Occasionally
◯ Sometimes ◯ Quite a lot
◯ Most of the time

Genetics

DNAmt depletion
- ◯ Yes
- ◯ No
- ◯ Untested

If yes, specify tissue studied
- ◯ Muscle
- ◯ Blood
- ◯ Urine
- ◯ Buccal mucose
- ◯ Other

If yes, specify % of heteroplasmy ______________________

DNAmt deletion
- ◯ Yes
- ◯ No
- ◯ Untested

If yes, specify
- ◯ Single
- ◯ Multiple

DNAmt sequencing
- ◯ Negative
- ◯ Positive
- ◯ Untested

Molecular diagnosis
- ◯ DNAmt
- ◯ DNAn

Single deletion DNAmt
- ◯ Yes
- ◯ No

If yes specify size ______________________

If yes, specify location ______________________

DNAmt (select positive study)

◯ mtRNA Leu (MTTL2) ◯ mtRNA Ile (MTT1)
◯ mtRNA Lys (MTTK) ◯ mtRNA Ser (MTTS1) ◯ mtRNA Ser (MTTS2)
◯ mtRNA Trp (MTTW) ◯ mtRNA Gln (MTTQ)
◯ mtRNA Ala (MTTA) ◯ mtRNA Tyr (MTTY)
◯ mtRNAGlu (MTTE) ◯ mtRNA Cys (MTTC)
◯ mtRNA Asp (MTTD) ◯ mtRNA Thr (MTTT)
◯ mtRNA Phe (MTTF) ◯ mtRNA Pro (MTTP)
◯ mtRNA Met (MTTM) ◯ mtRNA Leu 1 MTTL1 ◯ mtRNA Val MTTV
◯ mtRNA Asp MTTN ◯ mtRNA Gly MTTG
◯ mtRNA Arg MTTR ◯ mtRNA His MTTH
◯ mtrRNA 12S MTRNR1
◯ mtrRNA 16S MTRNR2
◯ MTND1 ◯ MTND2
◯ MTND3 ◯ MTND4
◯ MTND4L ◯ MTND5
◯ MTND6 ◯ MTCYB ◯ MTCO1
◯ MTCO2 ◯ MTCO3 ◯ MT-ATP6
◯ MT-ATP8

DNAmt Mutation ________________________________

DNAmt heteroplasmy ________________________________

DNAn (select positive study)

◯ AARS2
◯ AASS
◯ ABAT
◯ ABCB6
◯ ABCB7
◯ ACAD8
◯ ACAD9
◯ ACADM
◯ ACADS
◯ ACADSB
◯ ACADSD
◯ ACADVL
◯ ACAT1
◯ ACO2
◯ ACSL4
◯ ADCK1
◯ ADCK2
◯ ADCK3
◯ ADCK4
◯ ADCK5
◯ AFG3L2
◯ AGK
◯ AGXT
◯ AIFM1
◯ AK2
◯ ALAS2
◯ ALDH18A1
◯ ALDH2
◯ ALDH4A1
◯ ALDH5A1
◯ ALDH6A1
◯ AMACR
◯ AMT
◯ APEX1
◯ APEX2
◯ ATAD3A
◯ ATAD3B
◯ ATP5A1
◯ ATP5B
◯ ATP5C1
◯ ATP5D
◯ ATP5E
◯ ATP5F1
◯ ATP5G1
◯ ATP5G2
◯ ATP5G3
◯ ATP5H
◯ ATP5I
◯ ATP5J
◯ ATP5J2
◯ ATP5L
◯ ATP5L2
◯ ATP5O
◯ ATP7B
◯ ATPAF1
◯ ATPAF2
◯ ATPIF1
◯ AUH
◯ BCKDHA
◯ BCKDHB
◯ BCL2
◯ BCS1L
◯ BOLA1
◯ BOLA2
◯ BOLA3
◯ BRIP1
◯ C10ORF2
◯ C12ORF65
◯ CARS2
◯ CBR4

- ◯ CIAPIN1
- ◯ CLPB
- ◯ CLPX
- ◯ COA5
- ◯ COQ10A
- ◯ COQ10B
- ◯ COQ2
- ◯ COQ3
- ◯ COQ4
- ◯ COQ5
- ◯ COQ6
- ◯ COQ7
- ◯ COQ9
- ◯ COX10
- ◯ COX11
- ◯ COX14
- ◯ COX15
- ◯ COX16
- ◯ COX17
- ◯ COX18
- ◯ COX19
- ◯ COX20
- ◯ COX4I1
- ◯ COX4I2
- ◯ COX5A
- ◯ COX5B
- ◯ COX6A1
- ◯ COX6A2
- ◯ COX6B1
- ◯ COX6B2
- ◯ COX6C
- ◯ COX7A1
- ◯ COX7A2L
- ◯ COX7A2P2
- ◯ COX7B
- ◯ COX7C
- ◯ COX8
- ◯ CPOX
- ◯ CPS1
- ◯ CPT1A
- ◯ CPT2
- ◯ CRBN
- ◯ CYC1
- ◯ CYCS
- ◯ CYP11A1
- ◯ CYP11B1
- ◯ CYP11B2
- ◯ CYP24A1
- ◯ CYP27A1
- ◯ CYP27B1
- ◯ DARS2
- ◯ DBT
- ◯ DDX28
- ◯ DGUOK
- ◯ DHODH
- ◯ DHX30
- ◯ DIABLO
- ◯ DLAT
- ◯ DLD
- ◯ DMGDH
- ◯ DNA2
- ◯ DNAJC19
- ◯ DNM1L
- ◯ DUT
- ◯ EARS2
- ◯ ECHS1
- ◯ ECSIT
- ◯ ELAC2
- ◯ EMC8
- ◯ ENDOG
- ◯ ERCC6

- ◯ ERCC8
- ◯ ETFA
- ◯ ETFB
- ◯ ETFDH
- ◯ ETHE1
- ◯ FARS2
- ◯ FASN
- ◯ FASTKD2
- ◯ FBXL4
- ◯ FDX1
- ◯ FDX1L
- ◯ FDXR
- ◯ FECH
- ◯ FEN1
- ◯ FH
- ◯ FOXRED1
- ◯ FXN
- ◯ GARS
- ◯ GATM
- ◯ GCDH
- ◯ GCSH
- ◯ GFER
- ◯ GFM1
- ◯ GFM2
- ◯ GK
- ◯ GLDC
- ◯ GLRX5
- ◯ GLUD1
- ◯ GRPEL1
- ◯ GRPEL2
- ◯ GSH
- ◯ GSR
- ◯ HADH
- ◯ HADHA
- ◯ HADHB
- ◯ HADHSC
- ◯ HARS2
- ◯ HAX1
- ◯ HCCS
- ◯ HIBCH
- ◯ HIGD1A
- ◯ HK1
- ◯ HLCS
- ◯ HMGCL
- ◯ HMGCS2
- ◯ HSCA
- ◯ HSCB
- ◯ HSD17B8
- ◯ HSD3B2
- ◯ HSPA1A
- ◯ HSPA9
- ◯ HSPD1
- ◯ HTRA2
- ◯ IARS2
- ◯ IBA57
- ◯ IBA57-HOMOLOG
- ◯ IDH2
- ◯ IDH3B
- ◯ ISCA1
- ◯ ISCA2
- ◯ ISCA2-HOMOLOG
- ◯ ISCU
- ◯ IVD
- ◯ KARS
- ◯ KIF1B
- ◯ LARS2
- ◯ LIAS
- ◯ LIG3
- ◯ LIPT1
- ◯ LIPT2
- ◯ LONP1

◯ LRPPRC
◯ LYRM4
◯ LYRM7
◯ MAOA
◯ MARS2
◯ MCCC1
◯ MCCC2
◯ MCEE
◯ MDP1
◯ MECR
◯ MFF
◯ MFN2
◯ MGME1
◯ MIP
◯ MLYCD
◯ MMAA
◯ MMAB
◯ MME4
◯ MME6
◯ MPC1
◯ MPG
◯ MPV17
◯ MRPL12
◯ MRPL3
◯ MRPL44
◯ MRPL48
◯ MRPS16
◯ MRPS22
◯ MRRF
◯ MSH2
◯ MT-ATP6
◯ MT-ATP8
◯ MT-CO2
◯ MT-CO3
◯ MT-COI
◯ MT-CYB
◯ MTERF
◯ MTFMT
◯ MTIF2
◯ MTIF3
◯ MT-ND1
◯ MT-ND2
◯ MT-ND3
◯ MT-ND4
◯ MT-ND4L
◯ MT-ND5
◯ MT-ND6
◯ MTO1
◯ MTPAP
◯ MTRF1
◯ MTRF1L
◯ MTRR
◯ MUT
◯ MUTYH
◯ NADK2
◯ NARS2
◯ NDUFA1
◯ NDUFA10
◯ NDUFA11
◯ NDUFA12
◯ NDUFA13
◯ NDUFA2
◯ NDUFA3
◯ NDUFA4
◯ NDUFA4L2
◯ NDUFA5
◯ NDUFA6
◯ NDUFA7
◯ NDUFA8
◯ NDUFA9
◯ NDUFAB1

◯ NDUFAF1
◯ NDUFAF2
◯ NDUFAF3
◯ NDUFAF4
◯ NDUFAF5
◯ NDUFAF6
◯ NDUFB1
◯ NDUFB10
◯ NDUFB11
◯ NDUFB2
◯ NDUFB3
◯ NDUFB4
◯ NDUFB5
◯ NDUFB6
◯ NDUFB7
◯ NDUFB8
◯ NDUFB9
◯ NDUFC1
◯ NDUFC2
◯ NDUFS1
◯ NDUFS2
◯ NDUFS3
◯ NDUFS4
◯ NDUFS5
◯ NDUFS6
◯ NDUFS7
◯ NDUFS8
◯ NDUFV1
◯ NDUFV2
◯ NDUFV3
◯ NEIL1
◯ NFS1
◯ NFU1
◯ NMT1
◯ NT5M
◯ NTHL1
◯ NUBPL
◯ NUDT1
◯ OAT
◯ OGG1
◯ OPA1
◯ OPA3
◯ OTC
◯ OXA1L
◯ OXCT1
◯ PANK2
◯ PARK7
◯ PARP1
◯ PARS2
◯ PC
◯ PCCA
◯ PCCB
◯ PCK2
◯ PDHA1
◯ PDHA2
◯ PDHB
◯ PDHX
◯ PDK1
◯ PDK2
◯ PDK3
◯ PDP1
◯ PDP2
◯ PDSS1
◯ PDSS2
◯ PDX1
◯ PET100
◯ PHB
◯ PHB2
◯ PIF1
◯ PINK1
◯ PNPT1

- ◯ POLDIP2
- ◯ POLG
- ◯ POLG2
- ◯ POLRMT
- ◯ PPOX
- ◯ PRODH
- ◯ PTRF
- ◯ PUS1
- ◯ RAD51
- ◯ RARS2
- ◯ RFT1
- ◯ RMND1
- ◯ RMRP
- ◯ RNASEL
- ◯ RPP14
- ◯ RRM2B
- ◯ SARDH
- ◯ SARS2
- ◯ SCO1
- ◯ SCO2
- ◯ SCP2
- ◯ SDHA
- ◯ SDHAF1
- ◯ SDHAF2
- ◯ SDHB
- ◯ SDHC
- ◯ SDHD
- ◯ SERAC1
- ◯ SHMT2
- ◯ SLC19A2
- ◯ SLC19A3
- ◯ SLC25A12
- ◯ SLC25A13
- ◯ SLC25A15
- ◯ SLC25A19
- ◯ SLC25A20
- ◯ SLC25A22
- ◯ SLC25A28
- ◯ SLC25A3
- ◯ SLC25A37
- ◯ SLC25A4
- ◯ SLC25A5
- ◯ SLC46A1
- ◯ SLC52A2
- ◯ SLC52A3
- ◯ SLC9A6
- ◯ SOD2
- ◯ SPG7
- ◯ SSBP1
- ◯ STAR
- ◯ SUCLA2
- ◯ SUCLG1
- ◯ SUCLG2
- ◯ SUOX
- ◯ SUPV3L1
- ◯ SURF1
- ◯ TACO1
- ◯ TARS2
- ◯ TAZ
- ◯ TFAM
- ◯ TFB1M
- ◯ TFB2M
- ◯ TIMM44
- ◯ TIMM50
- ◯ TIMM8A
- ◯ TK2
- ◯ TMEM70
- ◯ TMLHE
- ◯ TOP1MT
- ◯ TP53
- ◯ TPK1

	◯ TRMU<br>◯ TSFM<br>◯ TTC19<br>◯ TUFM<br>◯ TXN2<br>◯ TYMP<br>◯ UNG<br>◯ UQCC1<br>◯ UQCC2<br>◯ UQCR10<br>◯ UQCR11<br>◯ UQCRB<br>◯ UQCRC1<br>◯ UQCRC2<br>◯ UQCRFS1<br>◯ UQCRH<br>◯ UQCRHL<br>◯ UQCRQ<br>◯ VARS2<br>◯ VDAC1<br>◯ VDAC2<br>◯ WARS2<br>◯ YARS2
Other genes	________________________________
Mode of inheritance	◯ Autosomic recessive<br>◯ Autosomic dominant<br>◯ X-linked recessive<br>◯ X-linked- dominant
Mutation 1	◯ Homozygous<br>◯ Heterozygous<br>◯ Hemizygous
cDNA change	
Protein change	
Pathogenecity	◯ Reported<br>◯ Not reported
Mutation 2	◯ Homozygous<br>◯ Heterozygous<br>◯ No second mutation
cDNA change	
Protein change	
Pathogenecity	◯ Reported<br>◯ Not reported
If new mutation, specify	◯ Family segregation<br>◯ Isolated fiber study (only in DNAmt mutation)<br>◯ Pathogenical based in bioinformatic analysis<br>◯ Other

Complementary Tests

Neurophtalmological tests	◯ Yes<br>◯ No
Electroretinography	◯ Normal<br>◯ Abnormal<br>◯ Not performed
Visual evoked potentials	◯ Normal<br>◯ Abnormal<br>◯ Not performed
Visual field	◯ Normal<br>◯ Abnormal<br>◯ Not performed
Fundus oculi	◯ Normal<br>◯ Abnormal<br>◯ Not performed
Optical Coherence Tomography	◯ Normal<br>◯ Abnormal<br>◯ Not performed
Other paediatric tests	◯ Yes<br>◯ No
Brainstem auditory evoked potencial	◯ Normal<br>◯ Abnormal<br>◯ Not performed
Nerve conduction studies and electromyographic	◯ Normal<br>◯ Abnormal<br>◯ Not performed
Electroencephalogram	◯ Normal<br>◯ Abnormal<br>◯ Not performed
Muscle biopsy	◯ Yes<br>◯ No
Histological findings	☐ Normal muscular biopsy<br>☐ Inepescific abnormalities in muscular biopsy<br>☐ Ragged red fibers<br>☐ COX negative fibers<br>☐ Subsarcolemmal deposits of SDH<br>☐ Fiber atrophy<br>☐ Fiber prevalence<br>☐ Dystrophic features<br>☐ Total or total depletion of oxidative enzymes<br>☐ Lipid storage<br>☐ Aspecific myopathic changesçNeurogenic changes<br>☐ Glycogen accumulation<br>☐ Subsarcolemmal rims<br>☐ Strongly succinate dehydrogenase-reactie blood vessels

If ragged red fibers, please specify

- ◯ >5
- ◯ 5-10
- ◯ 10-20
- ◯ < 20

If COX negative fibers, please specify

- ◯ Isolated COX negatives fibers
- ◯ All FRR are COX negatives
- ◯ All muscular fibers are COX negative

Respiratory chain

- ◯ Normal
- ◯ Abnormal

If abnormal please specify

- ◯ Complex I deficiency
- ◯ Complex III deficiency
- ◯ Complex IV deficiency
- ◯ Complex V deficiency
- ◯ Complex I+III+IV deficiency

Biomarkers tested

- ◯ Yes
- ◯ No

Plasma/serum

Lactic (Highest value in mmol/L)

- ◯ Normal
- ◯ Abnormal

If abnormal, specify value ______________________

Lactate/Pyruvate ratio

- ◯ Normal
- ◯ Abnormal

Alanine

- ◯ Normal
- ◯ Abnormal

FGF21

- ◯ Normal
- ◯ Abnormal

GDF15

- ◯ Normal
- ◯ Abnormal

Others ______________________

Urine

Lactic

- ◯ Normal
- ◯ Abnormal

If abnormal, specify value ______________________

Krebs cyclemetabolites

- ◯ Normal
- ◯ Abnormal

3-methylglutaconic

- ◯ Normal
- ◯ Abnormal

Others ______________________

Cerebrospinal fluid

Proteins
- ◯ Normal
- ◯ Abnormal

Lactic (Highest value)
- ◯ Normal
- ◯ Abnormal

If abnormal specify value ________________________________

Other ________________________________

Other complementary test

____

Neuroradiological features
- ◯ Yes
- ◯ No

Please specify
- ☐ Normal
- ☐ Cortical atrophy
- ☐ Cortical malformations
- ☐ Subcortical atrophy
- ☐ White matter abnormalities
- ☐ Stroke-like lesions
- ☐ Laminar necrosis
- ☐ Basal Nuclei involvement
- ☐ Dentate nuclei involvement
- ☐ Thalami / Subthalamic involvement
- ☐ Pontine / mesencephalic tegmentum involvement
- ☐ periaqueductal region involvement
- ☐ Bulbar involvement
- ☐ Cerebellar atrophy
- ☐ Cysts or vacuolated lesions
- ☐ Calcifications
- ☐ Corpus callosum agenesis

Treatment

Coenzima Q10 (CoQ10), Ubiquinona
◯ Yes
◯ No

Ubiquinol
◯ Yes
◯ No

Ibedenona
◯ Yes
◯ No

L-carnitine
◯ Yes
◯ No

Bicarbonato sódico/citrato sódico
◯ Yes
◯ No

B1 Vitamine or tiamine
◯ Yes
◯ No

C Vitamine or Ascorbat
◯ Yes
◯ No

B2 vitamine or Riboflavine
◯ Yes
◯ No

Biotine
◯ Yes
◯ No

Folinic acid
◯ Yes
◯ No

L-arginine
◯ Yes
◯ No

-L-citruline
◯ Yes
◯ No

Creatine
◯ Yes
◯ No

Cisteine
◯ Yes
◯ No

E Vitamine
◯ Yes
◯ No

Lipoic acid
◯ Yes
◯ No

Timidine and citidine nucleosides
◯ Yes
◯ No

Other treatments

____
