# Supplementary figures and images for: The Genetic Landscape of Mitochondrial Diseases in Spain: A Nationwide Call
Source: Genes (Basel). 2021 Oct 9;12(10):1590. doi: 10.3390/genes12101590 (PMC8535857; doi:10.3390/genes12101590)

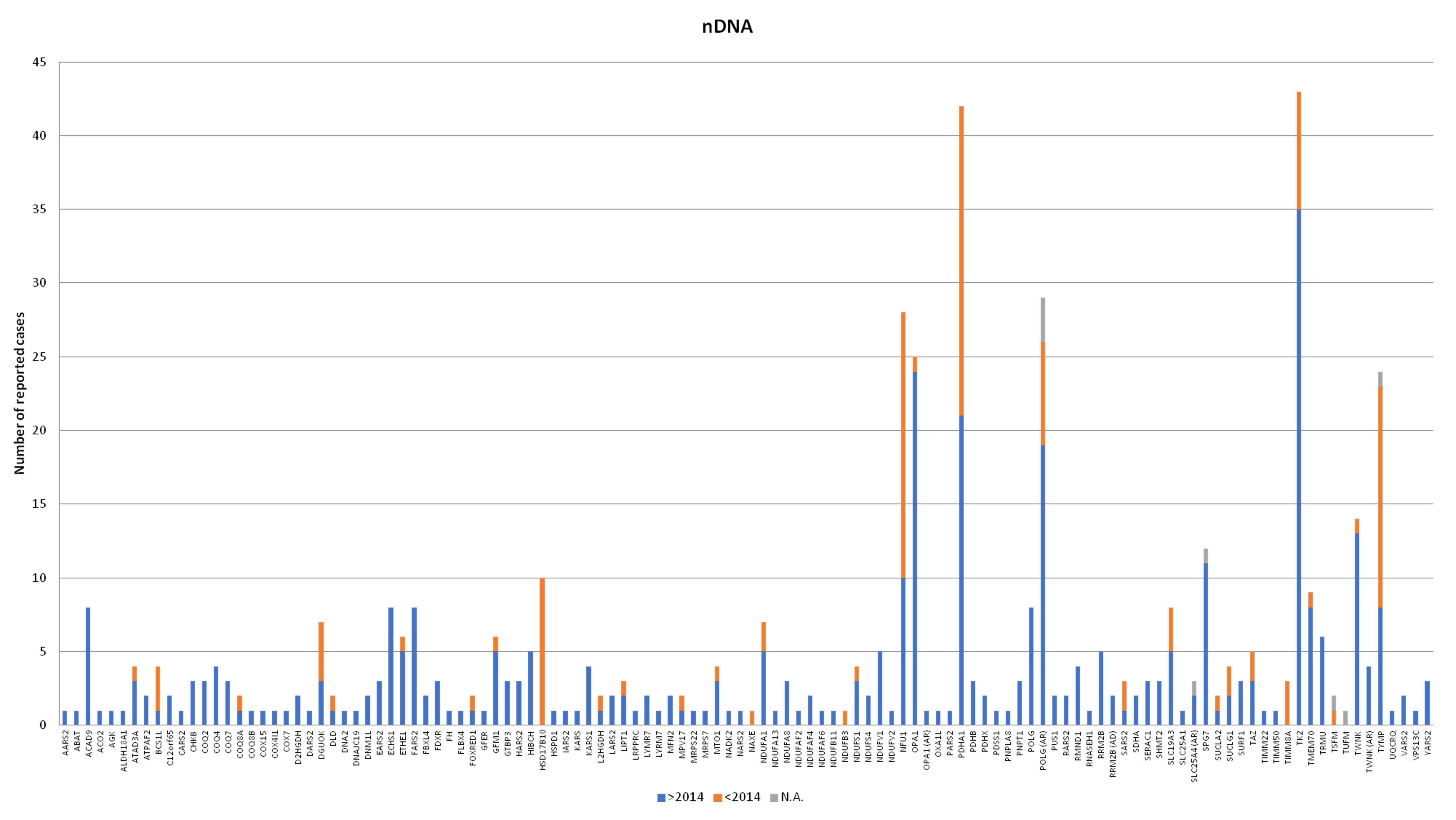

Supplement: Supplementary file 1 [file genes-12-01590-s001.zip › Supplementary Figure S1.tif]
